# Detection of anti-SARS-CoV-2 salivary antibodies in vaccinated adults

**DOI:** 10.3389/fimmu.2023.1296603

**Published:** 2023-11-07

**Authors:** Vitória Tavares Castro, Hélène Chardin, Juliana Amorim dos Santos, Gustavo Barcelos Barra, Grazielle Rodrigues Castilho, Paula Monteiro Souza, Pérola de Oliveira Magalhães, Ana Carolina Acevedo, Eliete Neves Silva Guerra

**Affiliations:** ^1^ Laboratory of Oral Histopathology, Faculty of Health Sciences, University of Brasilia, Brasília, DF, Brazil; ^2^ Department of Analytical, Bioanalytical Sciences and Miniaturization, École Supérieure de Physique et de Chimie Industrielles (ESPCI) de la Ville de Paris, Paris, France; ^3^ Unité de Formation et de Recherche d’Odontologie, Université Paris Cité, Paris, France; ^4^ Sabin Medicina Diagnostica Laboratory, Institute Sabin, Brasília, DF, Brazil; ^5^ Laboratory of Natural Products, Faculty of Health Sciences, University of Brasilia, Brasília, DF, Brazil

**Keywords:** COVID-19, SARS-CoV-2, saliva, antibodies, neutralizing antibodies, IgG, IgA, COVID-19 vaccines

## Abstract

Since the introduction of efficient anti-SARS-CoV-2 vaccines, the detection of antibodies becomes useful for immunological monitoring and COVID-19 control. Therefore, this longitudinal study aimed to evaluate the detection of SARS-CoV-2 antibodies in the serum and saliva of COVID-19-vaccinated adults. The study included 13 not vaccinated and 35 vaccinated participants with two doses of CoronaVac (Sinovac/Butantan) vaccine who subsequently received BNT162b2 (Pfizer-BioNTech) vaccine as a booster dose. Vaccinated participants donated saliva and serum in three different time points. Enzyme-linked immunosorbent assay was used for antibody detection. In our results, the serum neutralizing antibodies (NAb) were detected in 34/35 samples after second dose and in 35/35 samples one and five months after the booster dose. In saliva, NAb were detected in 30/35 samples after second dose and in 35/35 of samples one and five months after the booster dose. IgA was detected in 19/34 saliva samples after second dose, in 18/35 one month after the booster and in 30/35 five months after. IgG in saliva was detected in 1/34 samples after second dose, 33/35 samples one month after the booster dose and in 20/35 five months after. A strong correlation was found between IgG and neutralizing activity in saliva, and salivary IgA would be a sign of recent exposure to the virus. In conclusion, saliva can be suitable for monitoring antibodies anti-SARS-CoV-2 after vaccination. Heterologous vaccination contributed to increase anti-SARS-CoV-2 antibodies in the Brazilian health context. Complementary studies with large groups are mandatory to conclude the interest in following mucosal immunity.

## 1 Introduction

Since the COVID-19 outbreak, the development of efficient vaccines against severe acute respiratory syndrome coronavirus 2 (SARS-CoV-2) has been crucial for controlling the COVID-19 pandemic (1). Several vaccines were tested and developed at an unprecedented pace (2). CoronaVac (Sinovac/Butantan) uses inactivated whole virus (β-propiolactone-inactivated) and aluminum hydroxide as an adjuvant. The elementary vaccination regimen includes two vaccination doses with an interval of 2 to 4 weeks and the effectiveness against hospital admission is 85% and 80% against death (3).

Differently, Pfizer-BioNTech developed mRNA vaccines targeting the surface protein of SARS-CoV-2 (4). The BNT162b2 vaccine (Pfizer/BioNTech) contains a nucleoside-modified messenger RNA encoding the spike glycoprotein of SARS-CoV-2. This vaccine commits to reduce morbidity and mortality associated with SARS-CoV-2 infection by inducing Spike protein-specific antibodies providing protective immunity (5, 6).

Considering the current moment, continued monitoring and longitudinal studies are required to assess the duration of protection after COVID-19 vaccine shots over longer periods and to track population immunity (7). This control of the effectiveness of vaccination should help to decide the specific time point for everyone to receive a booster dose (8). A combination of heterologous COVID-19 vaccines can be used as a great strategy since heterologous prime-boost regimens may induce comparable or higher antibody titers than homologous prime-boost (9).

Serological testing was widely applied to identify exposure to the virus by detecting anti-SARS-CoV-2 specific antibodies and is now for vaccination follow-up (10). However, the invasive process needed for blood collection can limit the use of serological tests. Therefore, introducing saliva samples, composed of both gingival fluid and salivary secretion, can represent huge advantages as saliva collection is easy, non-invasive, and can be self-administrated with a lower risk of contamination (11, 12). Regarding daily mucosal antibody production, immunoglobulin A (IgA) production far exceeds the combined production of all other Ig isotypes. The majority of IgA is produced locally by plasma cells, densely distributed in the mucosal subepithelium, IgA is selectively transported and only traces of IgA in secretions originate from the circulation (13). Although the quantity of immunoglobulin G (IgG) secreted by the gingival fluid is limited compared to polymeric IgA from salivary glands, the advantages associated with saliva samples may improve the efficiency of monitoring and assessing vaccine responsiveness (14).

There is evidence that saliva is a valuable fluid in assessing immunity for various diseases. Some studies already point to a positive analysis of anti-SARS-CoV-2 antibodies in saliva, appearing to be a viable alternative to evaluate the effectiveness of vaccines (1, 15, 16). However, there is a lack of information about how antibody titers in saliva correlate with those measured using plasma serologic assays to identify anti-SARS-CoV-2-specific immunoglobulin activity.

Although mucosal immune responses have a critical role in protection against viral infections, they have been largely underestimated in the context of COVID-19, even with evidence of the important role of mucosal immunity. Since the SARS-CoV-2 virus first infects through the nasal passages and mouth, it is predicted that the first immune responses will occur through the mucosal immune system. The mucosal immune response induces secretory IgA antibodies in the secretions of the upper respiratory tract, tear fluid, and saliva (17). In a previous systematic review regarding the detection of anti-SARS-CoV-2 antibodies in saliva (18), none of the included studies were performed in Brazil, and none of them analyzed participants immunized with the CoronaVac vaccine. Most clinical studies focus on antibodies and cellular immunity in peripheral blood, while vaccine abilities to elicit a mucosal immune response is still under study (19). Few studies have explored mucosal immunity in individuals immunized with inactivated virus vaccines such as CoronaVac (20, 21). And none of them provided a longitudinal follow-up of the participants after the booster dose, with the view to monitoring salivary and serum antibodies and their time duration, as is the case of our study.

Thus, this longitudinal study aims to detect serum and saliva anti-SARS-CoV-2 antibodies in vaccinated adults with two doses of the CoronaVac vaccine who subsequently received the Pfizer vaccine as a booster dose.

## 2 Methods

This study was conducted according to the STROBE (Strengthening the Reporting of Observational Studies in Epidemiology) guideline and is in full accordance with ethical principles, including the World Medical Association Declaration of Helsinki, and has ethical approval from The Ethics Committee of the Faculty of Health Sciences, University of Brasília – UnB protocol number 48224221.2.0000.0030.

### 2.1 Participants

This study included 13 individuals not vaccinated and with no anti-SARS-CoV-2 antibodies detected in serum test, as a negative control, and 35 vaccinated participants with two doses of the CoronaVac (Sinovac/Butantan) vaccine who subsequently received the BNT162b2 (Pfizer-BioNTech) vaccine as a booster dose. The inclusion criteria were being over 18 years of age, not testing positive for the anti-SARS-CoV-2 NAbs serological test, and not presenting symptoms compatible with COVID-19.

**Figure 1:**
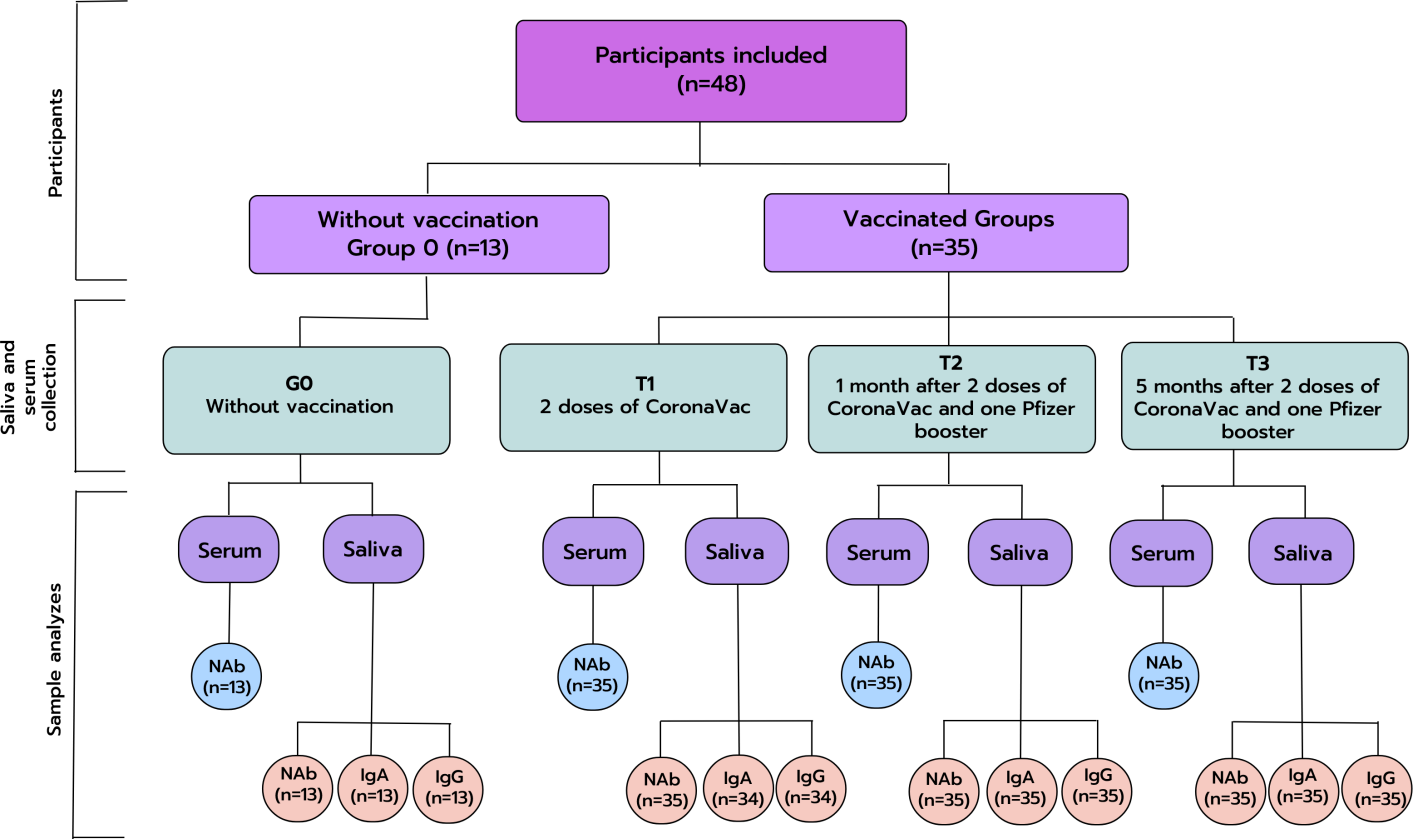
Flow diagram of participants and sample analyzes. IgA, Immunoglobulin A; IgG, Immunoglobulin G; NAb, Neutralizing Antibodies; TAb, Total Antibodies; n, number of participants.

All individuals donated serum and saliva samples, with a maximum interval of 7 days between saliva and blood collection, as described above. A cotton swab (Salivette^®^ Cortisol, SARSTEDT AG & Co, Germany) was used to collect stimulated saliva. **Figure 1** shows the flow diagram of participants and sample analyses. The participants were divided in groups, therefore:

**Without vaccination/negative control (n=13): group 0 (G0).**

- Individuals not vaccinated and without anti-SARS-CoV-2 antibodies detected in serum tests.
- Individuals donated saliva and serum once.

**Vaccinated group (n=35): Time points 1, 2, and 3.**

- **Time point 1 (T1)**: Individuals donated saliva and serum after two doses of the CoronaVac vaccine (Sinovac/Butantan); collection of saliva and serum were performed 2.5 months (median: 74 days; Q1: 61 days; Q3: 84 days) after the second dose, between July 07, 2021, to November 16, 2021.
- **Time point 2 (T2):** Individuals donated saliva and serum after two doses of CoronaVac vaccine (Sinovac/Butantan) and one booster dose of BNT162b2 (Pfizer/BioNTech) vaccine: collection of saliva and serum were performed one month (median: 36 days; Q1: 33 days; Q3: 41 days) after the booster dose, between October 26, 2021 to April 4, 2022.
- **Time point 3 (T3):** Individuals donated saliva and serum after two doses of the CoronaVac vaccine (Sinovac/Butantan) and one booster dose of BNT162b2 (Pfizer/BioNTech) vaccine: collection of saliva and serum were performed five months (median: 163 days; Q1: 156 days; Q3: 171 days) after the booster dose, between April 1, 2022, to July 30, 2022.

### 2.2 Demographic data collection

On the same day for serum and saliva collection, the participants answered a questionnaire about demographic data regarding being a health professional or not, age, and gender.

### 2.3 Collection and analysis of serum

Serum samples were stored at -20°C and all samples were processed at the Sabin Laboratory (Brasília, Federal District, Brazil).

### 2.4 Collection, transport, and preparation of saliva

All participants were instructed not to use oral hygiene products and not to consume alcoholic beverages, cigarettes, and/or food for at least 1 hour before saliva collection. The participants were instructed to chew a cotton swab (Salivette^®^ Cortisol, SARSTEDT AG & Co, Germany) for two minutes for the collection of stimulated total saliva. Whole saliva was collected containing products delivered from different oral cavity areas, such as minor salivary glands, gingival crevicular fluid, and microorganism subproducts.

Saliva was stored in a container with ice and transported to the laboratory within a maximum of 4 hours. Each swab containing saliva was centrifuged at 3,000 rpm for 5 minutes at 8°C. Then, the samples were stored at -80°C until processing. No inhibitors were used in the samples.

### 2.5 Neutralizing antibodies analyses

NAb were analyzed in all the serum and saliva samples at Sabin Laboratory (Brasilia, Federal District, Brazil). Serum was diluted according to the manufacturer’s instructions (1:9), and saliva was not diluted. Values higher or equal to 20 were considered positive and lower than 20 negative for serum. Values higher than 0 were considered positive, and lower or equal to 0 negative for saliva. Neutralizing activity was measured in optical density (OD), and reported as a percentage using the following equation according to the manufacturer’s instructions:


$$\text{X}=\frac{\text{(1-SampleOD)}}{\text{Negative Control}}\text{x}100=\%$$

### 2.6 Anti-SARS-CoV-2 IgA and IgG assays

Anti-SARS-CoV-2 IgA and IgG were both quantified in saliva samples by an ELISA test specific for IgA and an ELISA test specific for IgG, using a well plate coated with recombinant S protein antigen (Euroimmun Medizinische Laboradiagnostika, Luebeck, Germany, EI 2606-9601A EI 2606-9620A IgA, EI 2606-9601G EI 2606-9620G IgG), using an ELISA reader (EnSpire^®^ Multimode Plate Reader, PerkinElmer, USA), at Laboratory of Oral Histopathology (University of Brasilia, Federal District, Brazil) and Laboratory of Natural Products (University of Brasilia, Federal District, Brazil). Saliva was tested in the dilutions 1:101 (Manufacturer’s Instructions for blood), 1:50, 1:10, 1:5, and 1:2. The dilution 1:10 was chosen for IgA and 1:5 for IgG. Each sample was tested in triplicate and the average value was analyzed. Following the manufacturer’ instructions, values were then normalized for comparison with a calibrator. Results were evaluated by calculating the ratio between the extinction of samples and the extinction of the calibrator. Results are reported as the ratio between OD samples and OD calibrator:


$$\text{Ratio OD}=\frac{\text{sample absorbance OD}}{\text{test calibrator absorbance OD}}$$

### 2.7 Sensitivity, specificity, and accuracy values for salivary test

A 2x2 table was performed regarding the sensitivity, specificity, and accuracy of ELISA. For Nab assays values >0% were positive and values ≤0% were negative. Following the manufacturer’s instructions, results from IgA and IgG were considered positive if ratio OD ≥1.1, negative if<0.8, if ratio OD ≥0.8 and<1.1 were considered borderline samples and were excluded from this analysis. The reference test used for comparison was the results of NAb in serum. The sensitivity values were considered excellent when they were higher than 80%, 70-80% were considered good, 60-69% fair, and<60% poor. For specificity, >90% were excellent, 80-90% good, 70-79% fair, and<70% poor. Regarding accuracy >90% was excellent, 30-90% moderate, and<30% poor (22).

### 2.8 Statistics analyses

Descriptive and analytical statistics were performed using the GraphPad Prism software, version 9.3.0 (California, USA). The Shapiro-Wilk test was applied to assess data normality. Pearson’s correlation test was performed for parametric data, and Spearman’s correlation test for non-parametric data. For analyzing NAb titers in both serum and saliva samples, the T-test was used for parametric data and the Mann-Whitney test for non-parametric data to compare the negative control to vaccinated participants. To compare the three time points of the vaccinated group to the control group Kruskal-Wallis test was used followed by Dunn’s multiple comparisons post-test. In addition, to compare the values for the three different time points with each other for vaccinated group, the Friedman test for matched samples was used followed by Dunn’s multiple comparisons post-test.

## 3 Results

The negative control group (without vaccination - G0) was composed of 7/13 (53.85%) health professionals and the vaccinated group was composed of 19/35 (54.29%) health professionals. The average age for negative control was 29.23 years and for the vaccinated group 30.31 years, regarding sex 7/13 (53.85%) were female in negative control and 23/35 (65.71%) in the vaccinated group. In the negative control group, a total of 5 participants (38.46%) had some systemic disease while in the vaccinated group, 6 participants (17.14%) had some systemic disease. As for the use of systemic medication, 5 people (38.46%) used it in the negative control group and 9 participants (25.71%) in the vaccinated group. The groups composed of negative controls and vaccinated participants do not show statistically significant differences (**Supplementary Table 1**).

### 3.1 Neutralizing antibodies in serum and saliva samples

**Figure 2:**
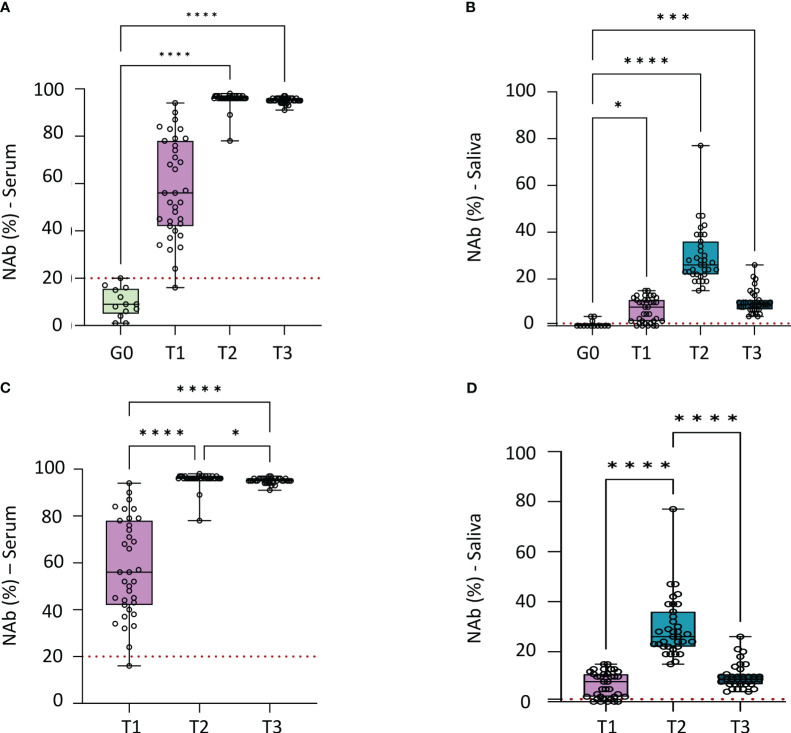
Median concentration of neutralizing antibodies in serum and saliva, the dotted red line indicates the cutt-off to each test. **(A)** Comparation of neutralizing antibodies (NAb) median in serum of negative control group to vaccinated group in each time point (p<0.0001). Statistical analysis: Kruskal-Wallis test. **(B)** Comparation of neutralizing antibodies (Nab) median in saliva of negative control group to vaccinated group in each time point (p<0.0001). Statistical analysis: Kruskal-Wallis test. **(C)** Comparation of neutralizing antibodies (NAb) median in serum for each time point of vaccinated individuals (p<0.0001). Statistical analysis: Friedman test. **(D)** Comparation of neutralizing antibodies (Nab) median in saliva for each time point of vaccinated individuals (p<0.0001). Statistical analysis: Friedman test. Graphpad Prism, version 9.5.0 (California, USA). NAb, of neutralizing antibodies. Group 0 (G0): without vaccination; T1: 2 doses of CoronaVac; T2: 1 month after 2 doses of CoronaVac and one booster with Pfizer; T3: 5 months after 2 doses of CoronaVac and one booster with Pfizer *p<0.05; ***p<0.0005; ****p<0.0001.

NAbs were detected in the saliva of 30/35 individuals vaccinated with two doses of Coronavac (T1). After the booster dose of Pfizer vaccine NAbs were detected in all individuals (T2 and T3, **Supplementary Table 2**). Anti-SARS-CoV-2 NAb values were higher in the vaccinated group in all time points compared to the non-vaccinated group in both serum (**Table 1**, **Figure 2A**) and saliva (**Table 1**, **Figure 2B**). NAbs increased one month after the Pfizer booster dose when compared with two doses of CoronaVac vaccine for serum (**Figure 2C**) and saliva (**Figure 2D**). Five months after the Pfizer booster dose, the neutralizing activity of the serum (**Figure 2C**) was maintained at the maximal value, whereas it began to drop in saliva (**Figure 2D**).

**Table 1:** Median and mean concentration of neutralizing antibodies in serum and saliva, IgA and IgG in saliva using ELISA method.

	Variables	Participants				
		G0 (n=13)	T1 (n=35)	T2 (n=35)	T3 (n=35)	p-value
**NAb Serum (%)**	**Median** **(Min-Max)**	9.00 (1-20)	56 (16-94)	96 (78-98)	95 (91-97)	<0.0001*
	**Mean** **(± SD)** **95% CI**	9.61 ( ± 6.06) 5.95-13.28	57.86 ( ± 20.74) 50.73-64.98	95.6 ( ± 3.34) 94.45-96.75	95.03 ( ± 1.17) 94.62-95.43	
**NAb Saliva (%)**	**Median** **(Min-Max)**	0 (0-4)	8 (0-15)	26 (15-77)	9 (4-26)	<0.0001*
	**Mean** **(± SD)** **95% CI**	0.77 ( ± 1.54) -0.16-1.7	6.77 ( ± 5.18) 4.99-8.55	29.5 ( ± 12) 25-34	10.2 ( ± 4.99) 8.5-11.12	
**IgA saliva (ratio OD)**	**Median** **(Min-Max)**	0.96 (0.44-3.78)	1.23 (0.20-4.25)	1.16 (0.40-8.16)	2.00 (0.33-6.79)	0.0008*
	**Mean** **(± SD)** **95% CI**	1.39 ( ± 1.15) 0.70-2.09	1.46 ( ± 1.01) 1.11-1.81	1.71 ( ± 1.65) 1.15-2.28	2.69 ( ± 1.72) 2.10-3.28	
**IgG saliva (ratio OD)**	**Median** **(Min-Max)**	0.31 (0.19-0.43)	0.32 (0.19-1.40)	3.08 (0.88-9.11)	1.20 (0.53-3.86)	<0.0001*
	**Mean** **(± SD)** **95% CI**	0.31 ( ± 0.07) 0.27-0.35	0.38 ( ± 0.21) 0.30-0.45	3.08 ( ± 1.63) 2.52-3.64	1.44 ( ± 0.76) 1.18-1.70	
**Days between vaccines last dose and saliva donation**	**Median** **(Min-Max)**	NA	74.00 (36-133)	36 (25-68)	163 (152-198)	<0.0001*
	**Mean** **(± SD)** **95% CI**	NA	74.51 ( ± 26.5) 65.41-83.62	38.54 ( ± 9.99) 35.11-41.97	165.51 ( ± 11.51) 161.60-169.50	

CI, Confidence Interval; G0, group 0; NA, Not Available; NAb, neutralizing antibodies; OD, Optical Density; SD, Standard deviation. Analytical statistics: Friedman test (*p<0.05) for matched non-parametric sample comparing T1, T2 and T3.Group 0 (G0): without vaccination; T1, Time point 1; T2, Time point 2; T3, Time point 3. Group 0: without vaccination; T1: 2 doses of CoronaVac; T2: 1 month after 2 doses of CoronaVac and one booster with Pfizer; T3: 5 months after 2 doses of CoronaVac and one booster with Pfizer.

### 3.2 Spike protein-specific IgA detection in saliva samples

**Figure 3:**
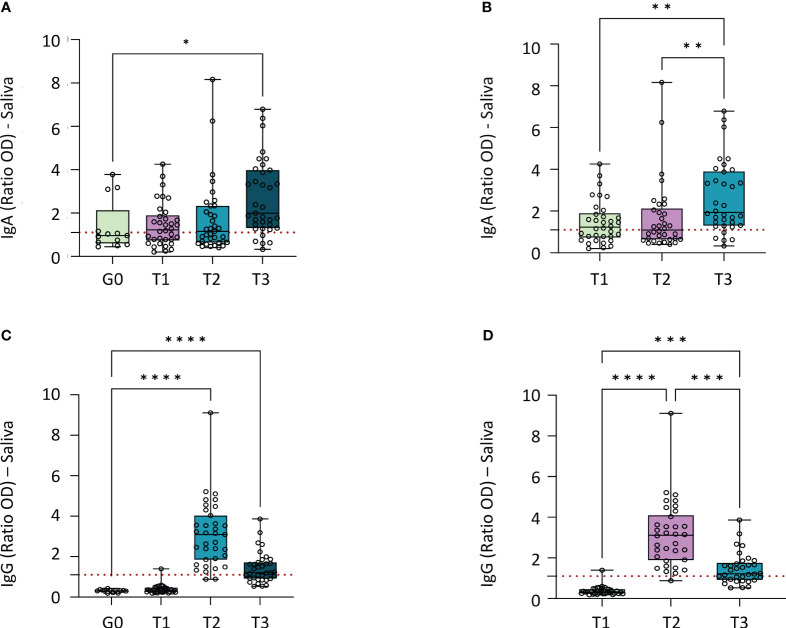
Median of IgA and IgG antibodies detected in saliva by ELISA method (Ratio OD). **(A)** Comparation of IgA median of negative control group to vaccinated group in each time point (p=0.0013). Statistical analyses: Kruskal-Wallis test. **(B)** Comparation of IgA median for each time point for vaccinated individuals (p=0.0008). Statistical analyses: Friedman test. **(C)** Comparation of IgG median of negative control group to vaccinated group in each time point (p<0.0001). Statistical analysis: Kruskal-Wallis test. **(D)** Comparation of IgG median for each time point for vaccinated individuals (p<0.0001). Statistical analyses: Friedman test. Graphpad Prism, version 9.5.0 (California, USA). IgA, Immunoglobulin A; IgG, Immunoglobulin G; OD, Optical Density. Group 0 (G0): without vaccination; T1: 2 doses of CoronaVac; T2: 1 month after 2 doses of CoronaVac and one booster with Pfizer; T3: 5 months after 2 doses of CoronaVac and one booster with Pfizer *p<0.05; **p<0.005; ***p<0.0005; ****p<0.0001.

Spike protein-specific IgAs were detected in the saliva of 4 out of the 13 in group 0. In the vaccinated group, the antibodies were detected in 19/34 individuals in T1, 18/35 in T2, and 30/35 in T3 (**Supplementary Table 2**). Negative controls had the lowest median of detected IgA antibodies (ratio OD= 0.96), classified as “limit” by the manufacturer’s instructions (**Figure 3A**). The vaccinated group had a positive median value in T1, T2, and T3, using the manufacturer’s instructions as a reference. After two doses of CoronaVac, the IgA ratio OD median was 1.23; one month after the Pfizer booster dose, the IgA ratio OD median was 1.16; and after five months, this value increased to 2.00 (p= 0.0008) (**Table 1**, **Figure 3C**). The only time statistically different from control was T3 (**Figure 3A**). When comparing vaccinated group in the three different time points, T3 was the only time statistically different (**Figure 3B**).

### 3.3 Spike protein-specific IgG detection in saliva samples

Spike protein-specific IgGs were not detected in the saliva of group 0. In T1, only one individual out of 34 was positive for spike protein-specific IgG. In T2, 33/35 were positive, and 20/35 individuals were still positive in T3 (**Supplementary Table 2**). Groups 0 and T1 had similar medians (ratio OD=0.31 and ratio OD=0.32, respectively) and T1 was the only group not statistically different from control (**Figure 3C**); this value increases in T2 (ratio OD= 3.08) and decreases in T3 (ratio OD=1.20), although it remains higher than the values of the group 0 and T1 (**Table 1**). When comparing vaccinated group in the three different time points, all the time points were statistically different from each other (**Figure 3D**).

### 3.4 Correlation of salivary IgA and IgG with neutralizing activity in saliva

**Figure 4:**
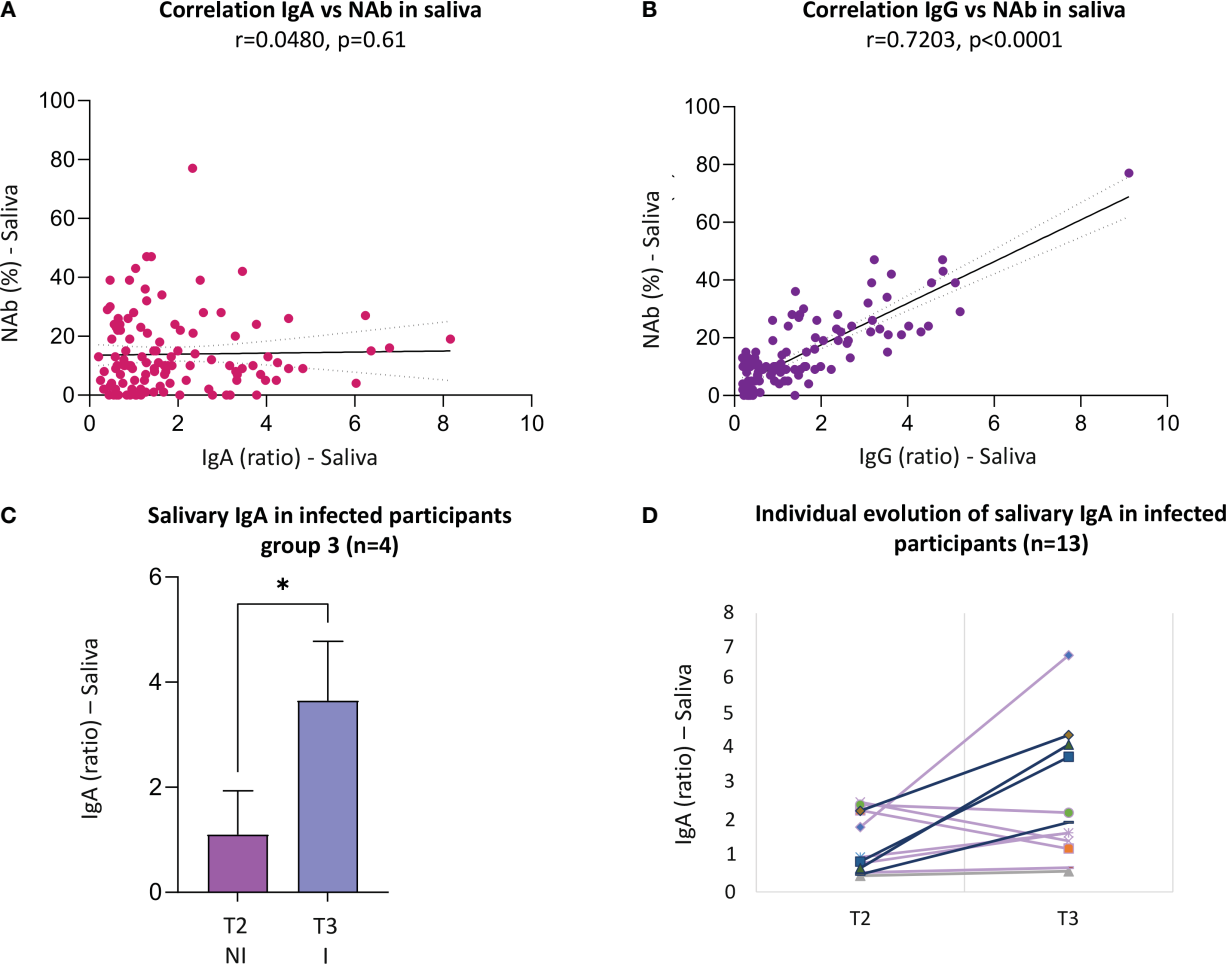
Significant correlations between different types of antibodies in saliva and IgA titers of infected participants. **(A)** Correlation IgG vs NAb in saliva for all the samples, there is a Strong correlation (n=117 pairs, r=0.72, p<0.0001). **(B)** Correlation IgA vs NAb in saliva for all the samples, the is no significant correlation (n=117 pairs, r=0.048, p=0.61). **(C)** Compartion of IgA titers of infected participants in T3 before and after infection (n=4), paired t test for parametric data (p=0.011*); **(D)** Individual evolution of salivary IgA in infected participants (n=13), purple lines are non-recent infected participants and lines in blue are the recent infected participants. Statistical analysis: For non-parametric data Spearman’s correlation. Graphpad Prism, version 9.5.0 (California, USA). Ab, antibodies; IgG, immunoglobin G; IgA, immunoglobin A; NAb, neutralizing antibodies; I, infected; NI, not infected; r, correlation coefficient. T2: 1 month after 2 doses of CoronaVac and one booster with Pfizer; T3: 5 months after 2 doses of CoronaVac and one booster with Pfizer *p<0.05.

In saliva, no significant correlation was found between IgA titers and the neutralizing activity at any time of collection (r=0.048, p=0.61) (**Figure 4A**, **Supplementary Table 3**). However, a strong significant correlation was found between IgG and neutralizing activity in saliva (r= 0.7203, p=<0.0001) (**Figure 4B**, **Supplementary Table 3**).

### 3.5 Comparison between infected and not infected by SARS-CoV-2

Since this is a longitudinal study, some vaccinated participants were infected by SARS-CoV-2 during the follow-up. In total, 9/35 participants were infected by SARS-CoV-2 one month after the booster dose (T2), and four other individuals were infected after five months (T3). A comparison was made between the antibody values to check if there was a statistical difference between individuals infected and not infected by SARS-CoV-2.

In T2 and T3, the values of NAbs were similar for infected and not infected participants with no statistical difference for serum or saliva. The same was observed for IgA and IgG in saliva (**Supplementary Table 4**, **supplementary figure 1**).

When analyzing the antibodies of the infected participants comparing before and after infection by SARS-CoV-2, an increase in the IgA titers after the infection was observed (**Figures 4C, D**). In T2, nine participants were infected, and IgA titers rose from 1.29 to 1.52 (paired t-test, p=0.446). Subsequently, 4 other participants were infected in T3 and the IgA titers rose from 1.09 to 3.65 (paired t-test, p=0.011), with a statistically significant difference between before and after infection. When IgAs were analyzed in all 13 infected participants, values varied from 0.78 (T1) to 0.93 (T2) and 1.84 (T4) (Friedman test, p=0.0458).

### 3.6 Sensitivity, specificity, and accuracy for salivary tests

The salivary test for detecting NAbs using ELISA (n=118) method showed excellent sensitivity (95%), fair specificity (77%), and excellent accuracy (93%) (**Supplementary Table 5**).

The sensitivity, specificity, and accuracy for the salivary NAb test using ELISA were also performed for the samples separately by groups. The specificity remained the same (76.9%) for all groups. T1 presented a great sensitivity (85.7%) and accuracy (83.3%) but was lower than the other groups and groups vaccinated with three doses (T2 and T3), which demonstrates an excellent sensitivity (100%) and accuracy (93.7%) (**Supplementary Table 5**).

The salivary test for IgA detection had a sensitivity of 72%, specificity of 55.5%, and accuracy of 70.5% when all the vaccinated groups were compared to group 0. When the parameters were analyzed separately for each group, the salivary test for IgA presented a specificity of 55.5% for all groups. The highest values of sensitivity and accuracy were for T3 (sensitivity= 88.2%, accuracy= 81.3%), followed by the values for T1 (sensitivity= 63.3%, accuracy= 61.5%) and the lowest values were for T2 (sensitivity= 62%, accuracy= 60.5%).

The salivary test for IgG detection had a sensitivity of 58.6%, specificity of 100%, and accuracy of 63.8% when all vaccinated time points were compared to group 0. For IgG detection in each group, the specificity was 100% for all groups. The highest sensitivity and accuracy were observed for T2 (sensitivity= 100%, accuracy= 100%), followed by T3 (sensitivity= 80%, accuracy=: 86.8%). The lowest values were for T1 because IgG were detected only in one of 34 samples (sensitivity: 2.9%, accuracy: 29.7%).

## 4 Discussion

The detection of salivary antibodies in anti-SARS-CoV-2 vaccinated individuals not only in serum but also in saliva is in accordance with previous results in the literature, confirming that salivary tests might be an alternative method to monitor the level of population immunity against SARS-CoV-2 (1, 23–25). All these studies have accessed antibodies from mRNA-based vaccines, whereas our study evaluated salivary antibodies in adults vaccinated with the non-mRNA-based-vaccine-CoronaVac, and after receiving a booster dose of Pfizer vaccine in three longitudinal time points.

After two doses of the CoronaVac vaccine, we found that the neutralizing activity of the serum was highly variable among individuals. Approximately a half of the individuals displayed a neutralizing activity in saliva, associated with low titers of spike protein-specific IgG in saliva and a low specificity of the IgG salivary test. Added to the previous conflicting studies of Chan et al., 2021 (21) in nasal secretion and Ortega et al., 2022 (20) in saliva, our results suggest that these tests are not appropriate to evaluate the diversity of the antibody response to inactivated virus vaccines but are solely built to assess the efficiency of the mRNA vaccines to induce a strong antibody response to the receptor binding domain of the spike protein. So, after a booster dose with the Pfizer/BioNTech vaccine, NAbs increased in saliva and serum. In serum, the maximal neutralizing activity was reached at one month and still present after five months. However, in saliva, the neutralizing activity decreased between one and five months, even though it was still detectable after five months. Moreover, a strong correlation was observed between the titer of salivary spike protein-specific IgG and NAb in saliva. This result agrees with Darwich et al., 2022 (26), in which salivary anti-SARS-CoV-2 specific antibodies IgG decreased in saliva three months after BNT162b2 vaccination. In addition, the fact that the maximal neutralizing activity was maintained at five months in the serum does not necessarily mean that the IgG titer is maintained, but only that there is enough IgG to totally inhibit the binding of a defined quantity of antigen-antibody complexes. Besides that, the test for salivary IgG presented an excellent accuracy of 100% one month after the BNT162b2 Pfizer booster dose. Thus, these high correlation and specificity could indicate that salivary anti-SARS-CoV-2 IgG comes from a serum exudate and saliva is suitable to follow such vaccination inducing high levels of IgG (18, 27).

Concerning the spike protein-specific salivary IgA titers, our results show a great heterogeneity in each group, the number of salivary IgA-negative individuals was similar between the non-vaccinated group and the group vaccinated with the CoronaVac only or after one Pfizer vaccine booster dose. Moreover, no correlation was observed between the salivary IgA titers and the neutralizing activity. These results, added to previous studies (18, 26, 28), suggest that the titer of salivary IgA does not reflect the efficiency of the vaccination but is more likely due to the exposure to the virus. Indeed, such intramuscular vaccines are dedicated to the induction of a strong immune response in the lymph nodes but are not adapted to a full mucosal immune stimulation. Consequently, they do not induce sterilizing immunity in the upper airway (29, 30). Furthermore, the systemic and mucosal IgA compartments are mutually independent, varying among antibodies structure, function, maturation, distribution, and antigenic specificity. These characteristics may explain the non-efficacy of the intramuscular vaccine for induction of mucosal immunity (31). Although intramuscular vaccines do not induce mucosal immunity in individuals previously uninfected by the virus, they can collaborate with the production of mucosal antibodies in previously infected individuals (32, 33). The induction of mucosal antibodies would prevent initial acquisition of the virus and reduce virus transmission. In this regard intranasal vaccines could present advantages (33).

As previously reported (1, 20, 34–37), the fact that high salivary IgA titers may be observed in individuals with no evidence, or no history of SARS-CoV-2 infection suggests that a strong mucosal immune response may prevent or limit systemic infection. Despite there was no significant difference in IgA between infected and non-infected participants, an increase in the IgA titers was observed after the infection. Considering our hypothesis that salivary IgA reflects the exposure to the virus, this result suggests that some people may be asymptomatic (and thus not identified as infected) with high salivary IgA titers, resulting in no difference in the average value among groups that were defined as infected or not on first criteria linked to the symptomatology. However, if we focus on newly symptomatic individuals only, a significant difference was found among salivary IgA production in T3. One reason to explain this difference may be the time of saliva collection. Indeed, when our collections were performed, the pandemic was not under control in Brazil, and the virus was contaminating many people at that time point. Moreover, the significant difference in the salivary IgA titers among T2 and T3 may be explained by the fact that the Omicron variant appeared in January 2022 in Brazil and is known to induce higher titers of secretory IgA than the previous variants (38). In summary, this increase in IgA in the saliva of vaccinated participants, especially in T3, could be explained by the Omicron variant that was circulating in Brazil during the period of saliva collection for T3.

Some methodological limitations of this study should be considered. First, the number of participants was small as this is a longitudinal study. Second, the tests for detecting anti-SARS-CoV-2 antibodies available on the market are designed for blood. In this work, we attempted to adapt it for saliva. Therefore, one of the difficulties was to define the cut-off values for salivary samples considered positive or negative. Third, tests were developed to evaluate the efficacy of RNA vaccines such as Pfizer, and the participants of this study took CoronaVac and a booster dose with Pfizer. Fourth, serum samples were only used to analyze NAbs but not IgA or IgG due to the unavailability of the samples during the time of the analysis. Fifth, the low sensitivity and specificity of the test for IgA detection may have affected/skewed the data and correlations.

## 5 Conclusion

There was an excellent sensitivity and accuracy for the salivary NAb test and excellent specificity for IgG salivary test, but the specificity for the salivary IgA was poor. The commercial tests available are powerful for mRNA-based vaccine follow-up regarding serum NAb, salivary NAb and salivary IgG, but not adapted to the CoronaVac vaccine. After CoronaVac vaccination, a single booster dose of mRNA vaccine is sufficient to maintain a full neutralizing activity against SARS-Cov2 virus until five months. Thus, the heterologous vaccination contributes to increase neutralizing anti-SARS-CoV-2 antibodies in the Brazilian health context. At last, our results suggest that the salivary IgA titer would be a sign of recent exposure to the virus which can be useful to follow virus circulation in epidemic situations. However, it is crucial to highlight that complementary studies with wider groups are mandatory to validate the findings of this study and to conclude on the interest to follow this mucosal immunity.

## Data availability statement

The datasets presented in this study can be found in online repositories. The names of the repository/repositories and accession number(s) can be found in the article/**Supplementary Material**.

## Ethics statement

The studies involving humans were approved by the ethics committee of the Faculty of Health Sciences, University of Brasília – UnB protocol number 48224221.2.0000.0030. The studies were conducted in accordance with the local legislation and institutional requirements. The participants provided their written informed consent to participate in this study.

## Author contributions

VC: Data curation, Formal Analysis, Investigation, Methodology, Resources, Software, Validation, Visualization, Writing – original draft, Conceptualization. HC: Conceptualization, Data curation, Formal Analysis, Investigation, Methodology, Resources, Supervision, Validation, Visualization, Writing – review & editing. JA: Conceptualization, Data curation, Formal Analysis, Investigation, Methodology, Software, Writing – review & editing. GB: Data curation, Formal Analysis, Investigation, Methodology, Validation, Writing – review & editing. GC: Data curation, Formal Analysis, Investigation, Methodology, Validation, Visualization, Writing – review & editing. PS: Data curation, Formal Analysis, Investigation, Methodology, Writing – review & editing, Validation. PM: Data curation, Formal Analysis, Investigation, Methodology, Validation, Writing – review & editing. AA: Conceptualization, Data curation, Formal Analysis, Investigation, Methodology, Resources, Supervision, Validation, Visualization, Writing – review & editing. EG: Conceptualization, Data curation, Formal Analysis, Funding acquisition, Investigation, Methodology, Project administration, Resources, Supervision, Visualization, Writing – review & editing.
