# Supplementary material for: Detection of anti-SARS-CoV-2 salivary antibodies in vaccinated adults
Source: Front Immunol. 2023 Nov 7;14:1296603. doi: 10.3389/fimmu.2023.1296603 (PMC10661372; doi:10.3389/fimmu.2023.1296603)
Supplement: Supplementary file 1 [file DataSheet_1.docx]

Supplementary Material

**Detection of anti-SARS-CoV-2 salivary antibodies in vaccinated adults**

Vitória Tavares Castro, Hélène Chardin, Juliana Amorim dos Santos, Gustavo Barcelos Barra, Grazielle Rodrigues Castilho, Paula Monteiro Souza, Pérola de Oliveira Magalhães, Ana Carolina Acevedo, Eliete Neves Silva Guerra

**Supplementary Table 1.** Description of demographic data based on participants records.

|  |  | Without vaccination participants  (n=13) | Vaccinated participants (n=35) | p-value |
| --- | --- | --- | --- | --- |
| Health professional | **No (%)** | 6 (46.15%) | 16 (45.71%) | >0.999 |
|  | **Yes (%)** | 7 (53.85%) | 19 (54.29%) |  |
| Sex | **Male (%)** | 6 (46.15%) | 12 (34.29%) | 0.871 |
|  | **Female (%)** | 7 (53.85%) | 23 (65.71%) |  |
| Age | **Median (Min-Max)** | 25 (20-53) | 25 (19-73) | 0.842 |
|  | **Mean (± SD)**  **95% CI** | 29.23 (**±**10.22) 23.06-35.06 | 30.31 (±13.75) 25.75-34.866 |  |
|  | **19 - 49 years** | 12 (92.30%) | 30 (85.71%) | 0.934 |
|  | **50 - 79 years** | 1 (07.69%) | 5 (14.29%) |  |

Abbreviations: CI – Confidence Interval; Max – Maximum; Min – Minimum; SD – Standard deviation. Analytical statistics: Mann-Whitney test (*p<0.05).

**Supplementary Table 2.** Proportions of individuals positive for NAb, IgA and IgG antibodies.

| **Participants** | **NAb** | | **IgA** | **IgG** |
| --- | --- | --- | --- | --- |
|  | **Serum** | **Saliva** | **Saliva** | **Saliva** |
| **Group 0** - Without vaccination | 0/13  (0%) | 3/13  (23.08%) | 4/13  (30.77%) | 0/13  (0%) |
| **Time point 1** - Vaccinated with 2 doses of CoronaVac | 34/35  (97.14%) | 30/35  (85.71%) | 19/34  (55.88%) | 1/34  (2.86%) |
| **Time point 2** - Vaccinated with 2 doses of CoronaVac and one booster dose with Pfizer – one month after the last dose | 35/35  (100%) | 35/35  (100%) | 18/35  (51.48%) | 33/35  (94.29%) |
| **Time point 3** - Vaccinated with 2 doses of CoronaVac and one booster dose with Pfizer – five months after the last dose | 35/35  (100%) | 35/35  (100%) | 30/35  (85.71%) | 20/35  (57.14%) |

Abbreviations: IgA, immunoglobulin A; IgG, imunoglobulin G; NAb; neutralizing antibodies; T1, Time point 1; T2, Time point 2; T3, Time point 3. Group 0: without vaccination; T1: 2 doses of CoronaVac; T2: 1 month after 2 doses of CoronaVac and one booster with Pfizer; T3: 5 months after 2 doses of CoronaVac and one booster with Pfizer.

**Supplementary Table 3**. Correlations of different types of antibodies.

| **Antibodies** | **Participants** | **Estatistic Correlation Analysis** | **R-value** | **95% confidence interval** | **P-value**  **(two-tailed)** |
| --- | --- | --- | --- | --- | --- |
| **NAb in saliva versus NAb in serum** | **Group 0** (n=13) | Spearman | -0.515 | -0.84 to 0.068 | 0.0746 |
|  | **T1** (n=35) | Spearman | -0.157 | -0.47 to 0.19 | 0.3663 |
|  | **T2** (n=35) | Spearman | 0.223 | -0.13 to 0.52 | 0.1977 |
|  | **T3** (n=35) | Spearman | -0.021 | -0.36 to 0.32 | 0.9011 |
|  | **All samples** (n=118) | Spearman | 0.7150 | 0.61 to 0.79 | <0.0001* |
| **IgA in saliva versus NAb in saliva** | **Group 0** (n=13) | Spearman | -0.3650 | -0.77 to 0.25 | 0.2214 |
|  | **T1** (n=34) | Spearman | -0.0848 | -0.42 to 0.27 | 0.6047 |
|  | **T2** (n=35) | Spearman | 0.1623 | -0.19 to 0.48 | 0.3516 |
|  | **T3** (n=35) | Spearman | 0.3233 | -0.02 to 0.59 | 0.0582 |
|  | **All samples** (n=117) | Spearman | 0.04778 | -0.14 to 0.23 | 0.6090 |
| **IgG in saliva versus NAb in saliva** | **Group 0** (n=13) | Pearson | -0.3710 | -0.76 to 0.23 | 0.2120 |
|  | **T1** (n=34) | Spearman | -0.1031 | -0.43 to 0.25 | 0.5619 |
|  | **T2** (n=35) | Spearman | 0.3631 | 0.024 to 0.62 | 0.0321* |
|  | **T3** (n=35) | Spearman | 0.5380 | 0.24 to 0.74 | 0.0009* |
|  | **All samples** (n=117) | Spearman | 0.7226 | 0.62 to 0.80 | <0.0001* |
| **IgG in saliva versus NAb in serum** | **Group 0** (n=13) | Spearman | 0.1570 | -0.45 to 0.66 | 0.6060 |
|  | **T1** (n=34) | Spearman | 0.3925 | 0.05 to 0.65 | 0.0238* |
|  | **T2** (n=35) | Spearman | 0.02728 | -0.32 to 0.37 | 0.8783 |
|  | **T3** (n=35) | Spearman | -0.0389 | -0.38 to 0.31 | 0.8243 |
|  | **All samples** (n=117) | Spearman | 0.8035 | 0.73 to 0.86 | <0.0001* |

Abbreviations: IgA - immunoglobulin A; IgG - immunoglobulin G; m – months; NAb - neutralizing antibodies, T1, Time point 1; T2, Time point 2; T3, Time point 3. Group 0: without vaccination; T1: 2 doses of CoronaVac; T2: 1 month after 2 doses of CoronaVac and one booster with Pfizer; T3: 5 months after 2 doses of CoronaVac and one booster with Pfizer.

**Supplementary Table 4.** Comparation between individuals infected by SARS-CoV-2 and not infected by SARS-CoV-2.

|  | Variables | Vaccinated with 3 doses after 1 month (T2, n=35) | | | Vaccinated with 3 doses after 5 months (T3, n=35) | | | |
| --- | --- | --- | --- | --- | --- | --- | --- | --- |
|  |  | **Infected by SARS-CoV-2 (n=9)** | **Not infected by SARS-CoV-2 (n=26)** | **p-value** | | **Infected by SARS-CoV-2 (n=13)** | **Not infected by SARS-CoV-2 (n=22)** | **p-value** |
| NAb Serum (%) | **Median (Min-Max)** | 96 (96-97) | 96 (78-98) | 0.885 | | 95 (93-96) | 95 (91-97) | 0.678 |
|  | **Mean (± SD) 95% CI** | 96.22 (±0.44) 95.93-96.51 | 95.38 (±3.87) 93.89-96.87 |  |  | 95 (±0.82) 94.56-95.44 | 95.04 (±1.36) 96.47-95.61 |  |
| NAb Saliva (%) | **Median (Min-Max)** | 29 (19-47) | 25 (15-77) | 0.234 | | 9 (4-18) | 9 (4-26) | 0.728 |
|  | **Mean (± SD) 95% CI** | 31.55 (±9.57) 25.3-37.8 | 28.81(±12.78) 23.9-33.72 |  |  | 9.92 (±3.80) 7.86-11.98 | 10.32 (±5.66) 7.96-12.68 |  |
| IgA Saliva (Ratio) | **Median (Min-Max)** | 1.86 (0.46-2.97) | 1.10 (0.40-8.16) | 0.540 | | 2.00 (0.59-6.79) | 2.13 (0.33-6.37) | 0.855 |
|  | **Mean (± SD) 95% CI** | 1.68 (±0.97) 0.93-2.42 | 1.72 (±1.85) 0.98-2.47 |  |  | 2.76 (±1.89)  1.62-3.90 | 2.65 (±1.65) 1.92-3.38 |  |
| IgG Saliva (Ratio) | **Median (Min-Max)** | 3.08 (0.88- 5.21) | 2.89 (0.88-9.11) | 0.697 | | 1.39 (0.66-2.68) | 1.18 (0.53-3.86) | 0.468 |
|  | **Mean (± SD) 95% CI** | 3.19 (1.59) 1.97-4.41 | 3.04 (1.68)  2.36-3.72 |  |  | 1.51 (0.67) 1.10-1.91 | 1.40 (0.82) 1.04-1.76 |  |

Abbreviations: CI – Confidence Interval; IgA – Immunoglobulin A; IgG – Immunoglobulin G; Max – Maximum; Min – Minimum; NA – Not Available; NAb – neutralizing antibodies; SD – Standard deviation.
Analytical statistics: Kruskal-Wallis test (*p<0.05). Vaccinated with 3 doses: two doses of CoronaVac (Sinovac/Butantan) + one booster dose of BNT162b2 (Pfizer-BioNTech).

**Supplementary Table 5.** Table 2x2 used for the calculation of sensitivity, specificity and accuracy.

1. Calculation of sensitivity, specificity and accuracy for salivary antibodies detection using ECLIA after 2 doses of CoronaVac for detection of salivary TAb.

| **ECLIA for detection of TAb (n=20)** | | |
| --- | --- | --- |
|  | Vaccinated (n=10) | Without vaccination (n=10) |
| Positive for TAb | 3 | 0 |
| Negative for TAb | 7 | 10 |

| **Parameters for TAb** | **ECLIA** |
| --- | --- |
| Sensitivity [a / (a + c)] | 30% |
| Specificity [(d / (b + d)] | 100% |
| Accuracy [(a+d) / (a + b + c + d)] | 65% |

2. Calculation of sensitivity, specificity and accuracy for salivary antibodies detection using ELISA for detection of salivary NAb.

| **ELISA for detection of salivary NAb** | | **Vaccinated** | **Without vaccination** |
| --- | --- | --- | --- |
| Vaccinated with 2 doses of CoronaVac (n=48) | Positive for NAb | 30 | 3 |
|  | Negative for NAb | 5 | 10 |
| Vaccinated with 3 doses 1 month after the third dose (n=48) | Positive for NAb | 35 | 3 |
|  | Negative for NAb | 0 | 10 |
| Vaccinated with 3 doses 5 months after the third dose (n=48) | Positive for NAb | 35 | 3 |
|  | Negative for NAb | 0 | 10 |
| General (n=118) | Positive for NAb | 100 | 3 |
|  | Negative for NAb | 5 | 10 |

| **Parameters for NAb** | **T1** | **T2** | **T3** | **General** |
| --- | --- | --- | --- | --- |
| Sensitivity [a / (a + c)] | 85.7% | 100% | 100% | 95% |
| Specificity [(d / (b + d)] | 76.9% | 76.9% | 76.9% | 76.9% |
| Accuracy [(a+d) / (a + b + c + d)] | 83.3% | 93.7% | 93.7% | 93% |

3. Calculation of sensitivity, specificity and accuracy for salivary antibodies detection using ELISA for detection of salivary IgA.

| **ELISA for detection of salivary IgA** | | **Vaccinated** | **Without vaccination** |
| --- | --- | --- | --- |
| Vaccinated with 2 doses of CoronaVac (n=39) | Positive for IgA | 19 | 4 |
|  | Negative for IgA | 11 | 5 |
| Vaccinated with 3 doses 1 month after the third dose (n= 38) | Positive for IgA | 18 | 4 |
|  | Negative for IgA | 11 | 5 |
| Vaccinated with 3 doses 5 months after the third dose (n=43) | Positive for IgA | 30 | 4 |
|  | Negative for IgA | 4 | 5 |
| General (n=102) | Positive for IgA | 67 | 4 |
|  | Negative for IgA | 26 | 5 |

| **Parameters for IgA** | **T1** | **T2** | **T3** | **General** |
| --- | --- | --- | --- | --- |
| Sensitivity [a / (a + c)] | 63.3% | 62% | 88.2% | 72% |
| Specificity [(d / (b + d)] | 55.5% | 55.5% | 55.5% | 55.5% |
| Accuracy [(a+d) / (a + b + c + d)] | 61.5% | 60.5% | 81.3% | 70.5% |

4. Calculation of sensitivity, specificity and accuracy for salivary antibodies detection using ELISA for detection of salivary IgG.

| **ELISA for detection of salivary IgG** | | **Vaccinated** | **Without vaccination** |
| --- | --- | --- | --- |
| Vaccinated with 2 doses of CoronaVac (n=47) | Positive for IgG | 1 | 0 |
|  | Negative for IgG | 33 | 13 |
| Vaccinated with 3 doses 1 month after the third dose (n= 46) | Positive for IgG | 33 | 0 |
|  | Negative for IgG | 0 | 13 |
| Vaccinated with 3 doses 5 months after the third dose (n= 38) | Positive for IgG | 20 | 0 |
|  | Negative for IgG | 5 | 13 |
| General (n=105) | Positive for IgG | 54 | 3 |
|  | Negative for IgG | 38 | 13 |

| **Parameters for IgG** | **T1** | **T2** | **T3** | **General** |
| --- | --- | --- | --- | --- |
| Sensitivity [a / (a + c)] | 2.9% | 100% | 80% | 58.6% |
| Specificity [(d / (b + d)] | 100% | 100% | 100% | 100% |
| Accuracy [(a+d) / (a + b + c + d)] | 29.7% | 100% | 86.8% | 63.8% |

Abbreviations: ECLIA – electrochemiluminescence; ELISA – enzyme-linked immunosorbent assay; IgA - Immunoglobulin A; IgG - Immunoglobulin G; NAb - Neutralizing antibodies; TAb – Total Antibodies, T1, Time point 1; T2, Time point 2; T3, Time point 3. Group 0: without vaccination; T1: 2 doses of CoronaVac; T2: 1 month after 2 doses of CoronaVac and one booster with Pfizer; T3: 5 months after 2 doses of CoronaVac and one booster with Pfizer.

**Supplementary figure 1**. Comparation between salivary antibodies of infected and not infected participants from T2 (saliva collected 1 month after 2 doses of CoronaVac and one Pfizer booster) and T3 (saliva collected 5 month after 2 doses of CoronaVac and one Pfizer booster). (A) Comparation between serum antibodies of infected and not infected participants for Nab concentration. (B) Comparation between salivary antibodies of infected and not infected participantes for NAb concentration. (C) Comparation between salivary antibodies of infected and not infected participants for IgA concentration. (D) Comparation between salivary antibodies of infected and not infected participants for IgG concentration. Statistical analysis: Mann-Whitney test for non-parametric data (*p<0.05). Graphpad Prism, version 9.5.0 (California, USA). Abreviations: Ab - antibodies; IgA - immunoglobulin A; IgG - immunoglobulin G; NAb - neutralizing antibodies; I - Infected; NI – Not Infected.

**
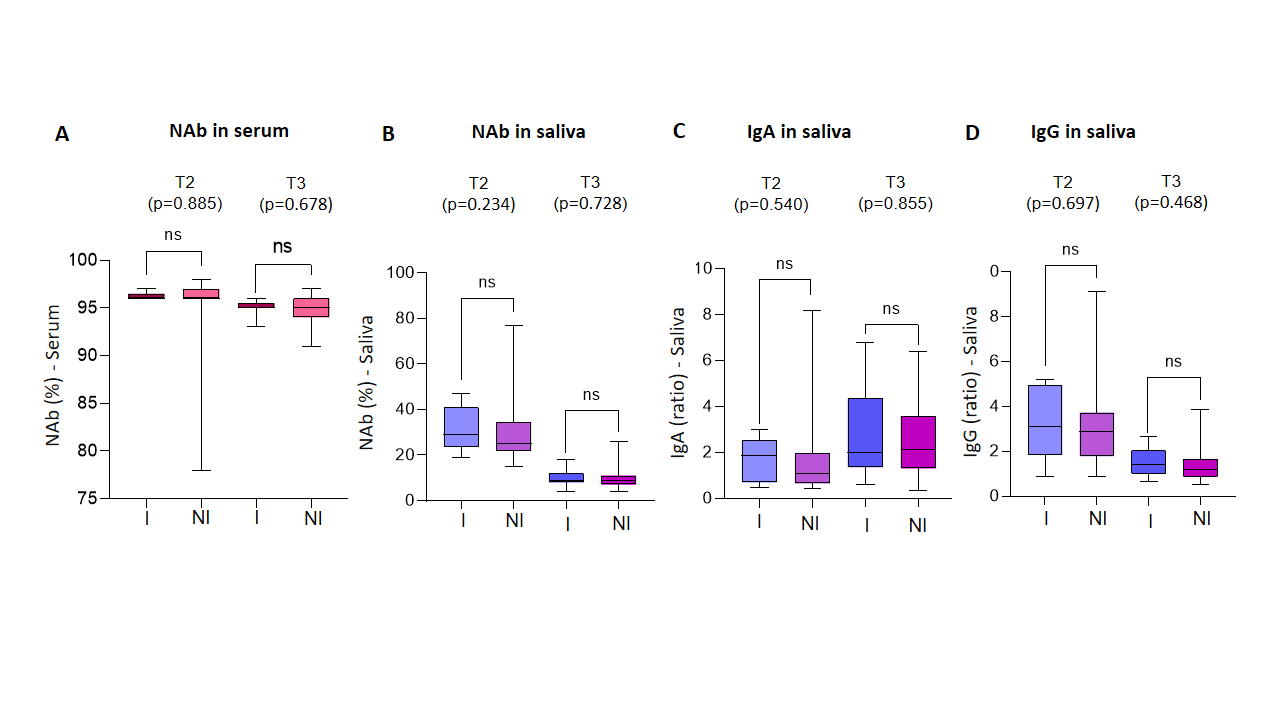
**
